# Layer-by-Layer Nanoarchitectonics: A Method for Everything in Layered Structures

**DOI:** 10.3390/ma18030654

**Published:** 2025-02-01

**Authors:** Katsuhiko Ariga

**Affiliations:** 1 Research Center for Materials Nanoarchitectonics (MANA), National Institute for Materials Science (NIMS), 1-1 Namiki, Tsukuba 305-0044, Ibaraki, Japan; ariga.katsuhiko@nims.go.jp; 2 Graduate School of Frontier Sciences, The University of Tokyo, 5-1-5 Kashiwanoha, Kashiwa 277-8561, Chiba, Japan

**Keywords:** layer-by-layer (LbL) assembly, living cell, metal–organic framework, molecular membrane, multilayer structure, nanoarchitectonics, nanoparticle, nanotechnology, thin film, 2D material

## Abstract

The development of functional materials and the use of nanotechnology are ongoing projects. These fields are closely linked, but there is a need to combine them more actively. Nanoarchitectonics, a concept that comes after nanotechnology, is ready to do this. Among the related research efforts, research into creating functional materials through the formation of thin layers on surfaces, molecular membranes, and multilayer structures of these materials have a lot of implications. Layered structures are especially important as a key part of nanoarchitectonics. The diversity of the components and materials used in layer-by-layer (LbL) assemblies is a notable feature. Examples of LbL assemblies introduced in this review article include quantum dots, nanoparticles, nanocrystals, nanowires, nanotubes, g-C_3_N_4_, graphene oxide, MXene, nanosheets, zeolites, nanoporous materials, sol–gel materials, layered double hydroxides, metal–organic frameworks, covalent organic frameworks, conducting polymers, dyes, DNAs, polysaccharides, nanocelluloses, peptides, proteins, lipid bilayers, photosystems, viruses, living cells, and tissues. These examples of LbL assembly show how useful and versatile it is. Finally, this review will consider future challenges in layer-by-layer nanoarchitectonics.

## 1. Introduction

In order to respond to a variety of social demands, including the resolution of energy issues [1,2,3,4,5], the management of environmental concerns [6,7,8,9,10], the handling of medical challenges [11,12,13,14,15], and the advancement of information technology [16,17,18,19,20], the scientific and technological communities must seriously engage with these issues. Material science occupies a pivotal position in this regard. The creation of material systems with enhanced functionality is identified as a key strategy for addressing these challenges. From the 20th century to the present, research fields related to material science have supported the creation of necessary functional materials. These disciplines encompass organic chemistry [21,22,23,24,25], inorganic chemistry [26,27,28,29,30], polymer chemistry [31,32,33,34,35], supramolecular chemistry [36,37,38,39,40], coordination chemistry [41,42,43,44,45], biochemistry [46,47,48,49,50], and other material sciences [51,52,53,54,55]. Among them, it has become evident that membranes on surfaces, membrane structures in solution media, and their multilayer structures have many beneficial properties [56,57,58,59,60]. These layered structures especially play an important role in various advanced functions [61,62]. Consequently, in many cases, layered structures are a primary focus in the creation of functional materials.

Two factors have been identified as being largely related to the development of functional layered structures. Firstly, the development of techniques for surface modification and bottom-up layer construction has been recognized as a key major factor. Surface chemistry and supramolecular chemistry have contributed to surface modification, the creation of membrane structures, and the creation of layered structures. These include self-assembled monolayer (SAM) [63,64], the Langmuir–Blodgett (LB) method [65,66], and layer-by-layer (LbL) assembly [67,68], which are based on various self-organization techniques rooted in supramolecular chemistry and surface science. Another development factor is the progress of nanotechnology, which has fundamentally changed the values of science and technology. The advent of nanotechnology has enabled the observation [69,70,71], manipulation [72,73,74,75], and evaluation of the physical properties [76,77,78,79,80,81] of nanostructures, down to the atomic and molecular level. Consequently, structures and functions that were previously only imaginable have become a reality, and the understanding of nanostructures through nanotechnology has been much advanced.

**Figure 1:**
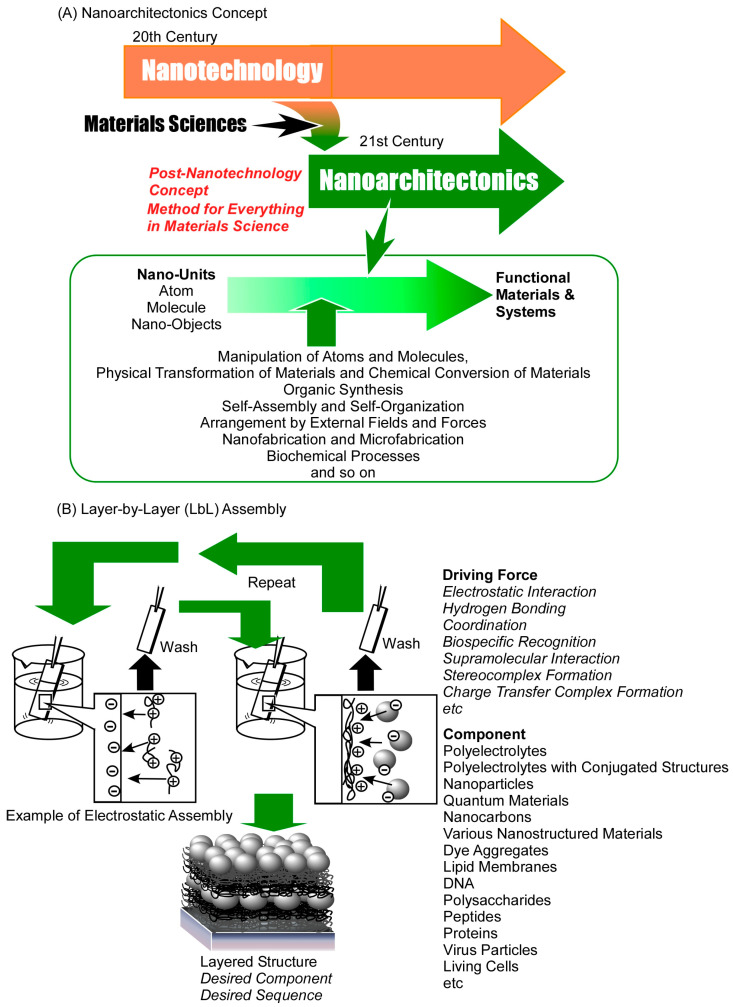
Outline of layer-by-layer nanoarchitectonics: (**A**) history and basic strategy of nanoarchitectonics; (**B**) typical assembling methods and varieties of components and driving forces.

The development of nanotechnology has not only led to the creation of layered structure materials, but it has also emphasized the significance of controlling various nanostructures and microstructures in the design of functions and the exploration of properties. The development of functional materials and the investigations in nanotechnology are ongoing endeavors. These fields interact inherently, yet there is a necessity to integrate them more proactively. The integration of these disciplines necessitates the development of a unifying concept that encompasses both nanotechnology and material science. While this may not constitute a wholly novel approach, the proposition of a novel concept is imperative to facilitate the convergence of science and technology, thereby ushering in a new era. Nanoarchitectonics, as a post-nanotechnology concept, are poised to fulfill this role (Figure 1A) [82]. Richard Feynman’s mid-20th century establishment of nanotechnology is often considered the foundation of this field [83,84], and Masakazu Aono’s proposal of nanoarchitectonics at the beginning of the 21st century is a significant development in this area [85]. Nanoarchitectonics is predicated on the knowledge of nanotechnology, and create functional materials. The concept of nanoarchitectonics involves the construction of functional materials from nano units, including atoms, molecules, and nanomaterials [86]. Nanoarchitectonics is not an unprecedented, completely new methodology, but rather an integrative concept that combines various fields.

In the domain of nanoarchitectonics, a range of processes is employed to construct functional materials from nano units [87,88]. These processes encompass a wide range of techniques, including the manipulation of atoms and molecules, the physical transformation of materials, and the chemical conversion of materials, known as organic synthesis. Additional processes include self-assembly and self-organization, arrangement by external fields and forces, nanofabrication and microfabrication, and biochemical processes. The construction of functional materials in nanoarchitectonics entails the judicious selection and combination of these processes. The combination of diverse processes in the construction of materials renders nanoarchitectonics a highly effective methodology for the fabrication of asymmetric and hierarchical structures [89]. The methodology of nanoarchitectonics is not limited to the particular type of material or its function, but is very general. Atomic and molecular composition underlies all materials, thus rendering nanoarchitectonics applicable to the full spectrum of materials. In a sense, then, it could be argued that, in a manner analogous to the ultimate goal of physics being the theory of everything [90], nanoarchitectonics is the method for everything in material science [91,92]. Consequently, nanoarchitectonics boasts a plethora of applications, irrespective of the material or intended use. A cursory review of publications bearing the term “nanoarchitectonics” in their title reveals its extensive contributions to diverse fields, including the synthesis of functional materials [93,94,95,96,97], the formation of specific structures [98,99,100,101,102], the exploration of physical phenomena and principles [103,104,105,106,107], basic biochemistry [108,109,110,111,112], catalysts [113,114,115,116,117], photocatalysts [118,119,120,121,122], electrochemical catalysts [123,124,125,126,127], sensors [128,129,130,131,132], biosensors [133,134,135,136,137], devices [138,139,140,141,142], environmental purification [143,144,145,146,147], fuel cells [148,149,150,151,152], solar cells [153,154,155,156,157], various batteries [158,159,160,161,162], supercapacitors [163,164,165,166,167], other energy applications [168,169,170,171,172], drug delivery [173,174,175,176,177], cell engineering [178,179,180,181,182], and medical applications [183,184,185,186,187].

As previously stated, the applications of nanoarchitectonics are diverse, and correspondingly, the structures that can be assembled also have huge variety. In this review paper, the focus is on the construction of layered structures, which account for a significant proportion of functional materials. As previously mentioned, the creation of functional materials by forming thin film layers on surfaces, creating molecular membranes, and creating structures with multiple layers of these has a wide impact. Consequently, the formation of layered structures accounts for a significant proportion of nanoarchitectonics [188,189]. In addition to methods for creating functional thin films using vacuum deposition [190,191], thin films and multilayer thin films are extensively developed using chemical wet processes. The creation of thin films using wet processes can be applied to a wide range of objects, including biological materials. These methods are more suitable for the nanoarchitectonics methodology of architecting materials.

Representative methods for producing thin films by wet processes include self-assembled monolayer (SAM) [192,193], the Langmuir–Blodgett (LB) method [194,195], and layer-by-layer (LbL) assembly [196,197]. Among these, LbL assembly is evaluated as a method that can be applied to a wider range of materials when producing multilayer structures (Figure 1B). The basic LbL assembly method uses electrostatic interactions [198,199]. In this method, thin films of nanometer-level thickness can be assembled in any layer number and layer sequence by using electrostatic interactions between positively and negatively charged substances. However, in LbL assembly, various other interactions can be used in place of electrostatic interactions. The driving force for LbL assembly can be various intermolecular interactions, including hydrogen bonding [200,201], coordination [202,203], biospecific recognition [204,205], supramolecular interactions [206], stereocomplex formation [207,208], and charge transfer complex formation [209,210]. The conventional method of LbL assembly involves the alternation of immersing a substrate in solutions of the film constituent materials. This method is the epitome of simplicity in nano-thin film production, requiring only a beaker and tweezers. Moreover, LbL assembly has been achieved through the utilization of spray [211,212] and spin coating [213,214] methodologies. Beyond the realm of LbL assembly on flat substrates, the application of LbL assembly on fine colloidal particles has emerged as a promising avenue [215,216]. The latter method has found application in the fabrication of microcapsules.

The most significant benefit of the LbL method is its versatility in fabricating layered structures with a wide variety of objects. For instance, materials exhibiting a surface charge can be utilized in the LbL method, which employs electrostatic interactions, and there is a substantial selection of such materials available. By expanding the range of driving forces for assembly, the scope for LbL assembly can be significantly expanded. The LbL method has been demonstrated to be applicable to a vast array of materials, including polyelectrolytes [217,218], polyelectrolytes with conjugated structures [219,220], nanoparticles [221,222], quantum materials [223,224], nanocarbons [225,226], various nanostructured materials [227,228,229], dye aggregates [230], lipid membranes [231,232], DNA [233,234], polysaccharides [235,236], peptides [237,238], proteins [239,240], virus particles [241,242], and living cells [243,244]. This extensive range of materials renders LbL assembly a promising candidate for a wide variety of applications in materials science. Consequently, layer-by-layer nanoarchitectonics emerges as a potentially comprehensive approach for the development of layered structures.

Due to the sheer diversity of the subject, it is practically impossible to introduce all of the characteristics and examples of LbL assembly in this single review. However, it is possible to demonstrate the wide range of possible nanoarchitectonics with examples. The examples here are grouped into 23 categories. In some cases, similar substances could be in separate categories. Therefore, they are classified according to their names in the literature of the substances to make it easier for the reader to sort them out. This review will demonstrate the breadth of applicability by presenting research examples of LbL assembly and related multilayer structure fabrication methods for a wide range of materials. This review has especially endeavored to provide examples from recent activities rather than historical research examples, as these may contain novel applications and concepts. It will introduce a wide range of examples of LbL assembly to demonstrate its breadth of applicability and versatility. Finally, this review will consider future challenges in layer-by-layer nanoarchitectonics.

## 2. LbL Materials

### 2.1. Quantum Dots

**Figure 2:**
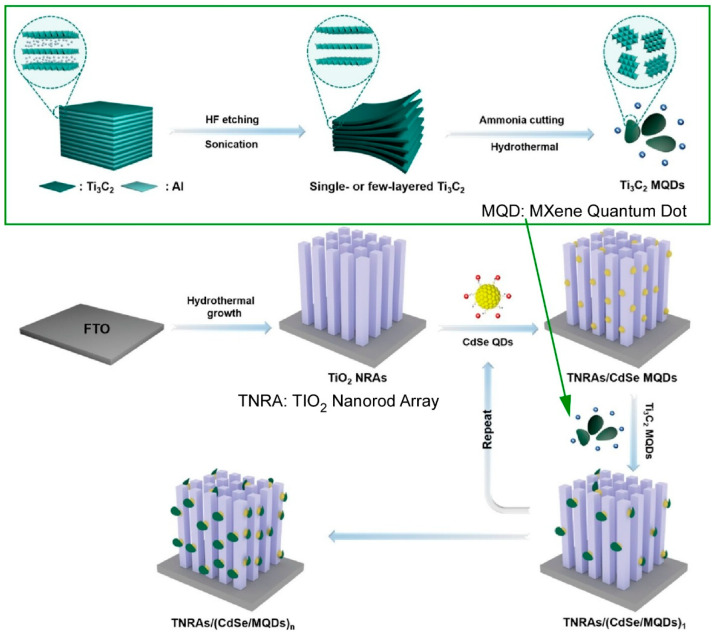
An electrostatic LbL assembly approach of oppositely charged transition metal chalcogenide quantum dot and MXene quantum dots in a metal oxide framework on TiO_2_ nanorod arrays. Reprinted with permission from [251]. Copyright 2024 Wiley-VCH.

Quantum materials are known to exhibit unique physical properties based on their nano-size. In addition, the organization of quantum materials into layered structures represents a fascinating challenge for functional development. LbL assembly of quantum materials, such as quantum dots, has also been considered [245,246]. For example, quantum dot (QD) colloidal nanocrystals have several physical property advantages, including a large optical absorption coefficient, a quantum confinement effect, abundant catalytic active sites, and a tunable electronic structure. Consequently, there has been significant interest in exploring their potential applications in fields such as solar energy conversion, where their unique properties could offer significant advantages [247,248,249,250]. However, the photoinduced charge carriers in quantum dots exhibit a very short charge lifetime, which poses a challenge to their stability. Therefore, the development of robust and stable quantum dot artificial photochemical systems with controllable functions is a highly desirable objective. Xiao and co-workers have reported an electrostatic LbL assembly approach of oppositely charged transition metal chalcogenide quantum dots (TMC QDs) and MXene quantum dots (MQDs) in a metal oxide (MO) framework (Figure 2) [251]. MOs/(TMCs QDs/MQDs)_n_ heterostructure photoanodes were nanoarchitectonicized by the LbL technique. Specifically, TiO_2_ nanorod arrays (TNRAs) were immersed in an aqueous solution of CdSe@AET quantum dots, resulting in the positive charging of 2-aminoethanethiol hydrochloride (AET)-modified CdSe quantum dots, thereby imparting positive charges to the surface of the TNRAs/CdSe quantum dots. The LbL assembly was then performed using negatively charged MXene quantum dots. The insertion of MXene quantum dots between the CdSe transition metal chalcogenide quantum dot layers has been shown to facilitate the photosensitizing effect of CdSe transition metal chalcogenide quantum dots and the electron capture ability of MXene quantum dots, thereby generating spatially cascaded electron transfer channels. The transition metal chalcogenide quantum dot layers act as light-harvesting antennas, and the MXene quantum dot layers act as electron capture mediators to relay cascaded electrons from the transition metal chalcogenide quantum dots to the metal oxide substrate. The spatially cascaded electron transfer channels generate spatially ordered tandem charge transport chains, which significantly improve the solar water oxidation efficiency of the photoanode. This layer-by-layer nanoarchitectonics of quantum dots may provide a route to strategically organize quantum dot-based charge transport for more efficient solar energy conversion.

### 2.2. Nanoparticles

**Figure 3:**
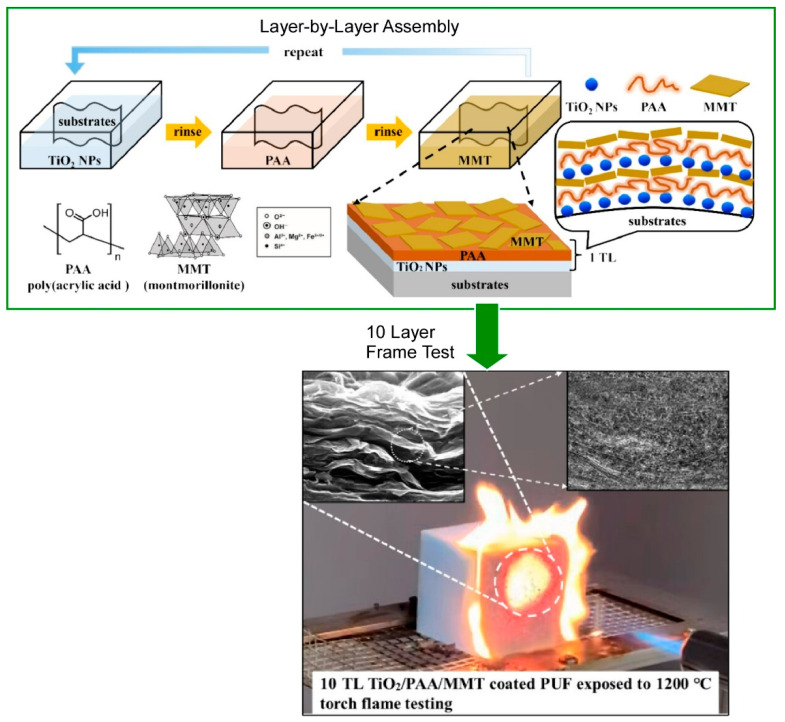
A flame retardant system of titanium dioxide (TiO_2_), montmorillonite (MMT), and poly(acrylic acid) (PAA) fabricated by LbL assembly: assembling method (**top**) and internal structures and flame retardant (**bottom**). Reprinted with permission from [258]. Copyright 2024 American Chemical Society.

The functionality and applications of nanoparticles are diverse [252,253,254,255]. A significant proportion of nanoparticles are charged, or can be modified to be charged, and as such, they have been extensively utilized as targets for LbL assembly [256,257]. Cho and colleagues have reported on a flame retardant system of titanium dioxide (TiO_2_), montmorillonite (MMT), and poly(acrylic acid) (PAA) fabricated by LbL assembly as a highly fire-resistant green nanocoating method [258]. As demonstrated in Figure 3, they nanoarchitectonicized a TiO_2_/PAA/MMT trilayer system, incorporating a polyelectrolyte layer between the metal nanoparticles and clay sheets. This layered design renders the coating to be significantly thicker and heavier. The incorporation of a polymer layer facilitates the incorporation of a greater quantity of clay and TiO_2_ nanoparticles, while preserving the integrity of the nanobrick wall structure. The incorporation of a polyelectrolyte between each layer of metal nanoparticles and clay platelets has also been demonstrated to enhance flame retardancy. The enhanced flame retardancy exhibited by the TiO_2_/PAA/MMT trilayer system can be attributed to the augmentation in char yield facilitated by the incorporation of the PAA polymer layer. The simplicity of the self-assembly approach via LbL assembly, the use of less toxic chemicals, and the greatly improved flame retardant properties of multilayer thin films may open new avenues for the protection of various objects against fire.

### 2.3. Nanocrystals

**Figure 4:**
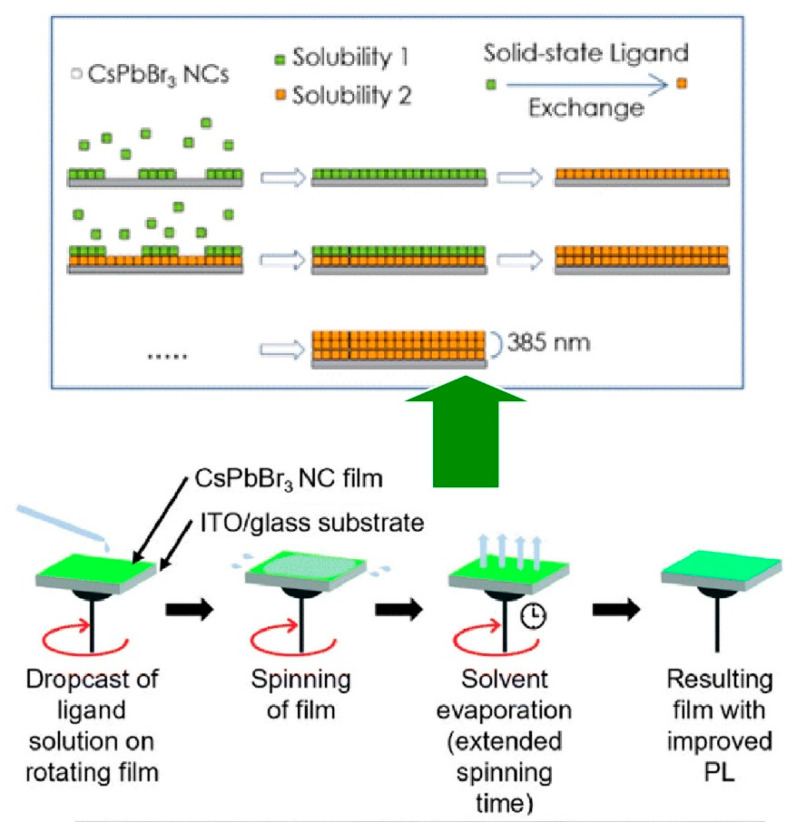
Solid-state ligand exchange of perovskite nanocrystal films, followed by layer-by-layer nanoarchitectonics by spin coating: plausible mechanism (**top**) and fabrication procedure (**bottom**). Reproduced under terms of the CC-BY license [265]. Copyright 2022 Royal Society of Chemistry.

Nanocrystals, defined as nano-objects with well-defined structures and crystal systems, represent attractive research targets for the development of advanced functions [259,260,261,262]. In particular, the thickness of nanocrystal films is a critical factor in the functionality of light-emitting diodes (LEDs), solar cells, and lasers. For instance, in the context of light-emitting diodes, the thickness of the light-emitting material film plays a pivotal role in determining the efficiency of the injected charge, which is crucial for the generation of bright electroluminescence. A similar relationship exists in solar cells, where the performance is determined by the amount of light absorbed, that is, the quantity of light absorbers within the device. Consequently, there is considerable value in exploring the layer-by-layer nanoarchitectonics of nanocrystals, using techniques such as LbL assembly, with respect to functionality development [263,264]. A notable example was reported by Francesco Di Stasio and his colleagues, who have demonstrated the LbL assembly method to produce high-quality films with finely controlled thickness using nanocrystals [265]. The possibility of ligand substitution in a solution of CsPbX_3_ (X = Cl, Br, or I) nanocrystals was exploited. Figure 4 demonstrates the solid-state ligand exchange of perovskite nanocrystal films, followed by layer-by-layer nanoarchitectonics by spin coating. The photoluminescence quantum yield values of CsPbBr_3_ nanocrystal films were enhanced by the introduction of didodecyldimethylammonium bromide and ammonium thiocyanate as new ligands on the nanocrystal surface. The deposition of the CsPbBr_3_ nanocrystal concentrated solution was performed in static mode on a spin coater, followed by spinning. This dynamic treatment can be achieved by depositing the ligand solution onto a rotating substrate and allowing it to move for an extended period. It was observed that the film thickness increases with increasing deposition times, and homogeneous films with thicknesses reaching 385 nm can be fabricated. It is further hypothesized that the facile LbL composite process of this method can be applied to other perovskite nanomaterials, thereby enabling layer-by-layer nanoarchitectonics of various layer combinations.

### 2.4. Nanowires

**Figure 5:**
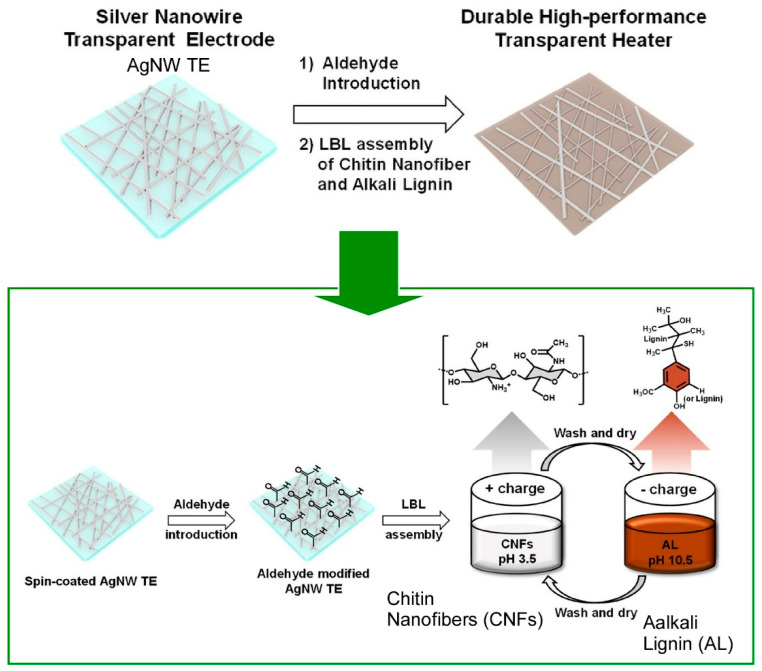
Fabrication strategy for Ag nanowire transparent electrodes, utilizing environmentally friendly materials such as chitin nanofibers (CNFs) and alkaline lignin (AL) through the LbL assembly method. Reprinted with permission from [272]. Copyright 2022 American Chemical Society.

Many one-dimensional nanostructures, including nanowires, nanorods, and nanofibers, exhibit various functionalities [266,267,268,269] with their structural elements, such as orientation, packing, and integration. Consequently, these one-dimensional nanomaterials have also been the focus of research in the field of LbL assembly [270,271]. In addition, the surface modification of nanowires through LbL assembly methods represents a promising approach for nanowire functionalization. For instance, Kim and colleagues have devised a straightforward and effective protection strategy for Ag nanowire transparent electrodes, utilizing environmentally friendly materials such as chitin nanofibers (CNFs) and alkaline lignin (AL), which are biorenewable and abundant natural polymers (Figure 5) [272]. This method was found to be effective in protecting silver nanowire-based transparent electrodes (AgNW TEs). It was demonstrated that the electrodes exhibited reduced adhesion to the substrate and enhanced thermal, optical, chemical, and electrical stability. In the study, chitin nanofibers and alkaline lignin were successively nanoarchitectonicized on an Ag nanowire transparent electrode through LbL assembly based on oppositely charged surfaces. The surfaces of chitin nanofibers and alkaline lignin are derived from amine and phenolic groups, respectively, and are oppositely charged, allowing electrostatic LbL assembly. The fabricated (CNF/AL)10Al-AgNW transparent electrode demonstrates considerable potential as a durable and effective transparent heater. This strategy can be regarded as a simple, efficient, and environmentally friendly layer-by-layer nanoarchitectonics approach for developing high-performance optoelectronic devices based on Ag nanowires.

### 2.5. Nanotubes

**Figure 6:**
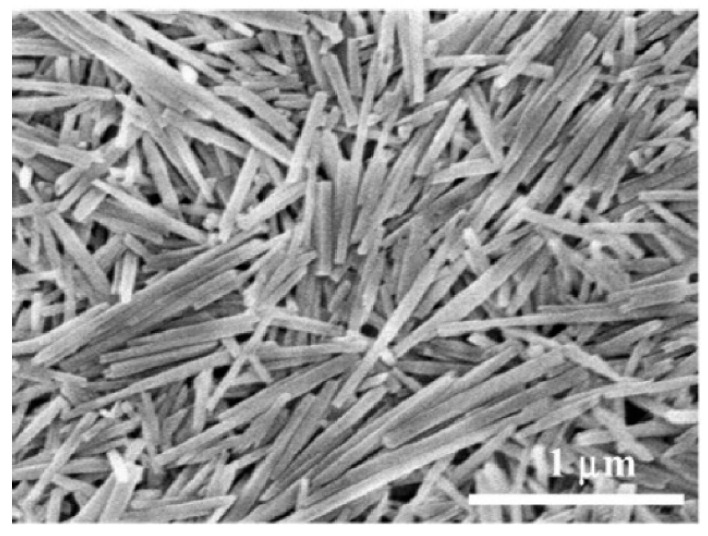
Halloysite nanotubes (Al_2_Si_2_O_5_(OH)_4_·nH_2_O) as clay nanoparticles composed of a rolled aluminosilicate sheet. Reprinted with permission from [281]. Copyright 2018 Wiley-VCH.

Nanotube materials, such as carbon nanotubes, have been extensively utilized in the fields of nanotechnology and functional materials research [273,274,275]. These nanotube materials have found application in LbL assembly and nanotube coating with LbL films [276,277]. The scope of nanotube materials extends beyond carbon nanotubes, encompassing clay nanotubes, such as halloysite nanotubes, which are also subjects of study [278,279,280]. Grunlan and colleagues have been investigating flame retardant coatings by an LbL assembly of halloysite nanotubes [281]; halloysite nanotubes (Al_2_Si_2_O_5_(OH)_4_·nH_2_O) are clay nanoparticles composed of rolled aluminosilicate sheets (Figure 6). In their research, they tried to develop a more environmentally friendly flame retardant for polyurethane foam. To this end, they layered halloysite nanotubes, stabilized with branched polyethyleneimine or polyacrylic acid, from aqueous suspension to create a multilayer nanocomposite coating. Generally speaking, halloysite is difficult to use in LbL assembly due to its poor dispersibility in water. To solve this problem, adsorption of polyelectrolytes, such as polyacrylic acid, is used. The adsorption of polyacrylic acid on the halloysite nanotube surface is facilitated by hydrogen bonding and van der Waals interactions, leading to enhanced stability of the suspension through negative electrostatic repulsion interactions. Additionally, anionic polyacrylic acid is primarily adsorbed on the lumen of the positively charged tubes, neutralizing the internal positive charge and increasing the overall negative particle zeta potential. Modification of halloysite with such polyelectrolytes allows for non-hazardous polymer-clay LbL assembly. The reported process enables the application of conformally composite coatings to three-dimensional foam structures, while preserving porosity and concomitantly, effecting a reduction in flammability. The hypothesis of the observed flame retardancy is substantiated by evidence that these composites form a physical barrier and dilute the gas phase through a dehydration mechanism, thereby reducing mass and energy transfer. The substantial decrease in both the peak heat release rate and the total smoke release, in addition to the self-extinguishing behavior exhibited by these halloysite-based multilayer films, serves to enhance the fire safety of polyurethane foams. It is anticipated that the incorporation of these halloysite-based composites will contribute to a reduction in fire-related injuries and fatalities by increasing escape times from burning homes and office buildings.

### 2.6. g-C_3_N_4_ and Graphene Oxide Materials

**Figure 7:**
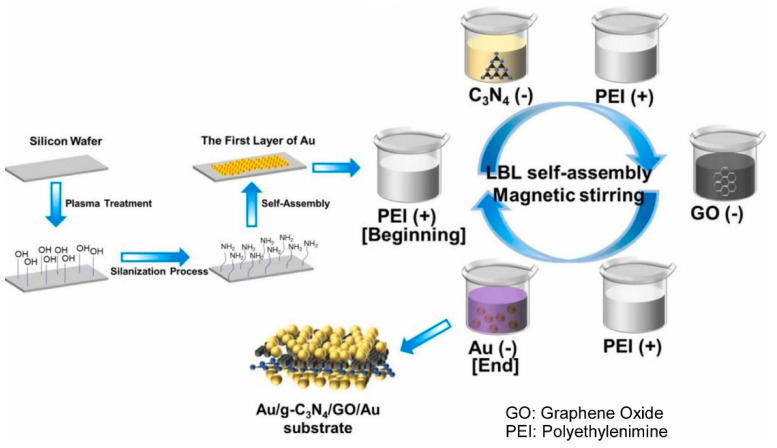
Fabrication of Au/g-C_3_N_4_/GO/Au hybrid nanofilms by LbL assembly using nanosheet-structured graphitic carbonitride (g-C_3_N_4_), graphene oxide (GO), and 40 nm gold nanoparticles (Au NPs). Reprinted with permission from [291]. Copyright 2024 Elsevier.

It is evident that 2D materials demonstrate a diverse array of functionalities [282,283,284]. Particularly noteworthy are materials such as g-C_3_N_4_ [285,286,287] and graphene oxide [288,289,290], which have garnered significant attention in recent years. The LbL assembly of these 2D materials has also been successfully executed. Guo, An, Han, and their colleagues fabricated Au/g-C_3_N_4_/GO/Au hybrid nanofilms by a straightforward LbL assembly technique, using nanosheet-structured graphitic carbonitride (g-C_3_N_4_), graphene oxide (GO), and 40 nm gold nanoparticles (Au NPs), as shown in Figure 7 [291]. This was used as a surface-enhanced Raman scattering (SERS) substrate. The first step in the synthesis of the Au/g-C_3_N_4_ material was to immerse an NH_2_-modified silicon wafer in a gold nanoparticle colloid solution, followed by washing with ultrapure water and drying. The silicon wafer was then immersed in a polyethyleneimine (PEI) solution, stirred, washed, and dried. It was then immersed in a g-C_3_N_4_ dispersion, stirred, and washed with ultrapure water before being dried once more. Subsequently, the LbL assembly of graphene oxide layer and gold layer was performed using a similar procedure. Au/g-C_3_N_4_ was immersed and stirred in PEI-1h/GO and PEI-1h/Au solutions to prepare the Au/g-C_3_N_4_/GO/Au substrate. The sensitivity of the substrate was found to be improved by the combination of the electromagnetic enhancement effect of gold nanoparticle aggregates and the chemical enhancement effect of graphene oxide and g-C_3_N_4_. As a demonstrative example, the detection of food colorings, erythrosine, carmine, and temptation red, was contemplated, and the food safety standard was successfully met. In this layer-by-layer nanoarchitectonics, the sheet-like g-C_3_N_4_ and graphene oxide played a crucial function of stabilizing the hot spots of gold nanoparticles. Such structure formation is expected to improve SERS performance for trace detection applications.

### 2.7. Nanosheet

**Figure 8:**
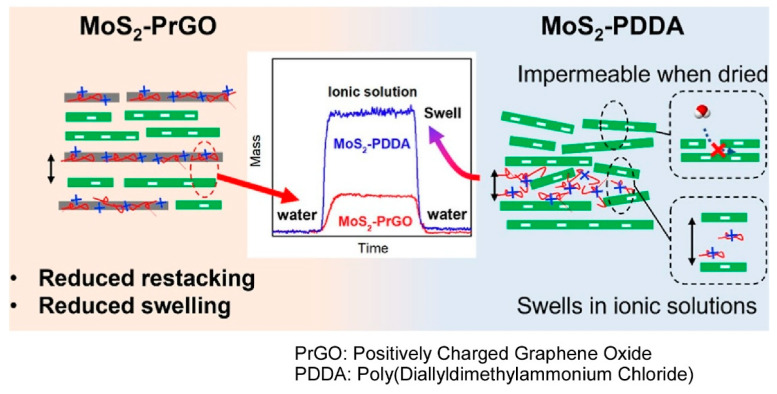
Comparisons of LbL assemblies of negatively charged molybdenum disulfide nanosheets (MoS_2_) with either positively charged graphene oxide (PrGO) nanosheets or a positively charged polymer (poly(diallyldimethylammonium chloride), PDDA). Reproduced under terms of the CC-BY license [297]. Copyright 2024 American Chemical Society.

A plethora of other 2D materials have been the subject of study in the context of nanosheets [292,293,294], and such nanosheets have also been identified as attractive targets for LbL assembly [295,296]. The process of the LbL assembly of oppositely charged materials frequently gives rise to the creation of electrically stabilized nanosheet–polymer structures. In contrast, Mi and co-workers have proposed that the use of 2D nanosheets with matching physical properties to both polyanions and polycations may lead to more ordered nanostructures, with better stability than nanosheet–polymer structures [297]. In order to draw parallels between nanosheet–nanosheet and nanosheet–polymer structures, negatively charged molybdenum disulfide nanosheets (MoS_2_) were LbL-assembled with either positively charged graphene oxide (PrGO) nanosheets or a positively charged polymer (poly(diallyldimethylammonium chloride), PDDA) (Figure 8). The assembly of PrGO, a nanosheet-based polycation, resulted in a significantly more aligned structure with MoS_2_ nanosheets, owing to the similarity in shape, size, and charge density. The restacking of MoS_2_ upon drying was more effectively controlled by positively charged graphene oxide compared to positively charged polymers. Consequently, MoS_2_-PrGO membranes exhibited enhanced stability under high ionic strength conditions and facilitated higher water flux with superior size exclusion. Specifically, the membranes composed of nanosheets with matching size, shape, and charge density exhibited a more aligned stacking structure, which reduced membrane swelling in high salt solutions, controlled restacking, and improved the separation performance. This exemplifies the pivotal role of compatibility among constituent materials in layer-by-layer nanoarchitectonics.

### 2.8. Zeolites

**Figure 9:**
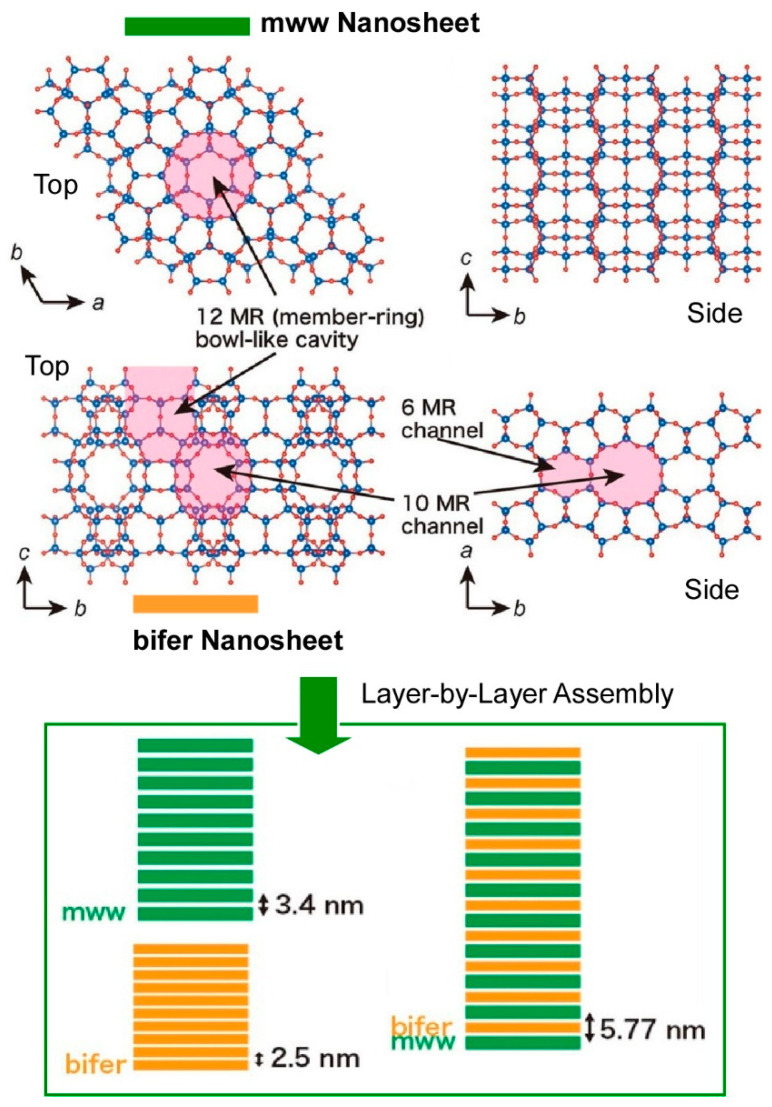
Electrostatic LbL assembly of two types of zeolite nanosheets with different porous structures, MWW topology (mww), and ferrierite-related structure (bifer) (Figure 9): zeolite structures (**top**) and possible LbL architectures (**bottom**). Reproduced under terms of the CC-BY license [301]. Copyright 2024 Wiley-VCH.

Porous materials have also attracted significant interest. Zeolites, for instance, possess distinctive porous structures that demonstrate molecular sieving properties and are extensively utilized as catalysts, adsorbents, and ion exchange resins [298,299,300]. Examples of the LbL assembly of such zeolites have been reported. Furthermore, some layered zeolites have the capacity to be exfoliated into molecularly thin 2D nanosheets, which feature unique porous structures and highly exposed active sites. Sasaki and co-workers have reported the electrostatic LbL assembly of two types of zeolite nanosheets with different porous structures, MWW topology (mww) and a ferrierite-related structure (bifer) (Figure 9) [301]. This method involves the alternate deposition of polycations and zeolite nanosheets, resulting in the growth of multilayer films with stacking distances of 2–3 nm. The repeated LbL process in the desired order allows for the design of various hierarchical membranes, such as multilayers and superlattice membranes, which cannot be realized by direct synthesis approaches. It is further noted that this methodology can be extended to different types of zeolites and a variety of other nanosheets, thus opening up the possibility of zeolite-based materials with engineered functions for various applications. The ultrathin, customized structures are believed to be suitable for advanced applications, such as highly accurate and sensitive sensors, ultrathin catalyst layers, adsorbents, and ion exchangers.

### 2.9. Nanoporous Carbon Materials

**Figure 10:**
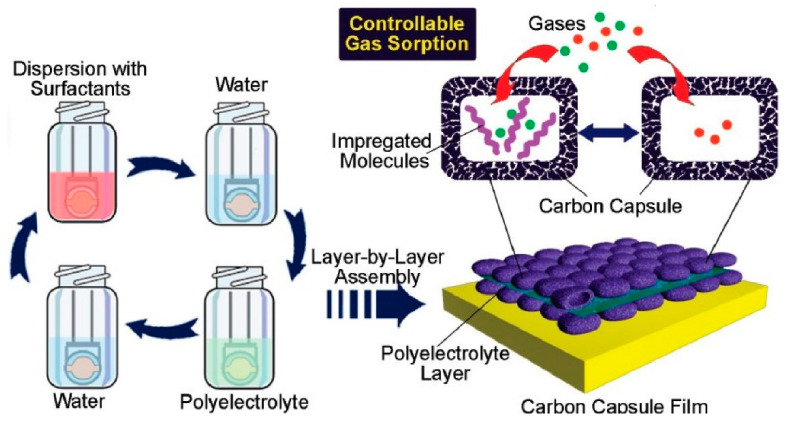
Fabrication of hierarchical thin films of carbon-based mesoporous capsules, also known as dual-pore carbon capsules, used as a sensor for volatile compounds. Reprinted with permission from [309]. Copyright 2009 American Chemical Society.

Mesoporous silica and various nanoporous materials possess unique functions due to their high specific surface area and controlled nano-space structures [302,303,304]. Research has been conducted on forming LbL assemblies in such porous structures [305,306], and there are also studies on organizing nanoporous materials themselves [307,308] by LbL assembly. In a seminal study, Ji, Yu, and co-workers synthesized dual-pore carbon capsules with mesoporous walls and macroscopic hollow cores, which were then stacked in layers by LbL assembly (Figure 10) [309]. The carbon capsules were surfactant-coated and stacked based on electrostatic interactions between oppositely charged polyelectrolytes. This process resulted in the formation of hierarchical thin films of carbon-based mesoporous capsules, also known as dual-pore carbon capsules. The resulting hierarchical carbon capsule films exhibited a significantly higher adsorption capacity in comparison to conventional layered materials, rendering them highly suitable for applications involving substance adsorption and sensing. Notably, the dual-pore carbon capsules demonstrated a stronger affinity for aromatic volatiles in comparison to aliphatic volatiles, which is attributable to the presence of stronger π−π interactions. Consequently, a sensor for aromatic volatiles was developed by fabricating this LbL film on a quartz crystal microbalance (QCM) sensor. Furthermore, sensors with altered adsorption selectivity can be designed by impregnating the carbon capsules with additional recognition components, thereby enabling the control of adsorption selectivity between aromatic and non-aromatic substances in the atmosphere, as well as between acids and bases. In the presence of functional groups, including water, acetic acid, ammonia, butylamine, aniline, and pyridine, the carbon capsules exhibited a pronounced affinity for aromatic guests, such as aniline and pyridine. The carbon capsule film impregnated with lauric acid demonstrated the strongest binding affinity for non-aromatic amines, followed by a secondary binding affinity for acetic acid. In contrast, the impregnation of the carbon capsule film with dodecylamine resulted in a strong selectivity for acetic acid. Given the stability of the carbon material in water, it is anticipated that this system can also be employed for the removal of toxic substances from water.

### 2.10. Sol–Gel Materials

**Figure 11:**
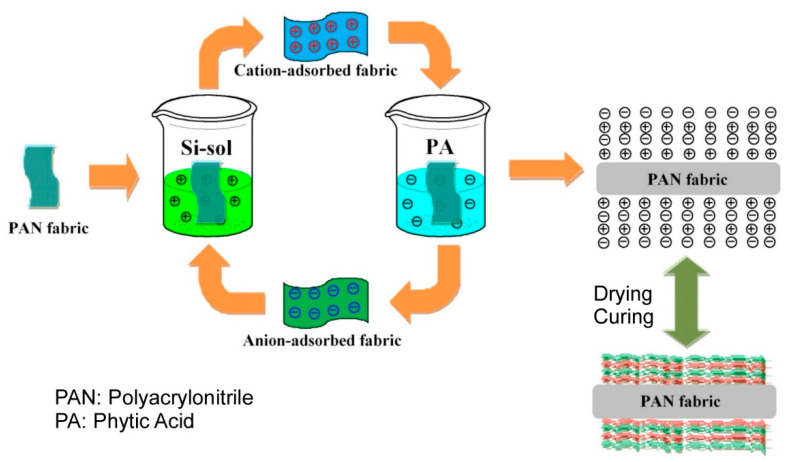
A method combining the sol–gel method and LbL assembly technology, where silica sol, synthesized by the sol–gel process, served as the cationic solution, while phytic acid was utilized in the LbL assembly as the anionic medium. Reproduced under terms of the CC-BY license [315]. Copyright 2018 MDPI.

The sol–gel technique has been extensively utilized in the fabrication of diverse inorganic, hybrid, and nanostructured materials [310,311,312]. There are also some research examples combining the surface sol–gel method with LbL assembly [313,314]. Ren et al. developed a method combining the sol–gel method and LbL assembly technology (Figure 11) [315]. The objective of this study was to enhance the flame retardant properties of polyacrylonitrile fabrics through the utilization of this novel method. In this study, silica sol, synthesized by the sol–gel process, served as the cationic solution, while phytic acid was utilized in the LbL assembly as the anionic medium. The resultant polyacrylonitrile fabrics treated with 10 bilayers of LbL coating demonstrated excellent flame retardancy. This outcome is attributed to the capacity of silica and phytic acid to impede the exchange of oxygen with the fabric. It is anticipated that these effects will be applicable to a range of functionalities, thus signifying that the combination of sol–gel and LbL assembly technology is a valuable method for imparting functionality to diverse materials.

### 2.11. Layered Double Hydroxides (LDH) with Dyes

**Figure 12:**
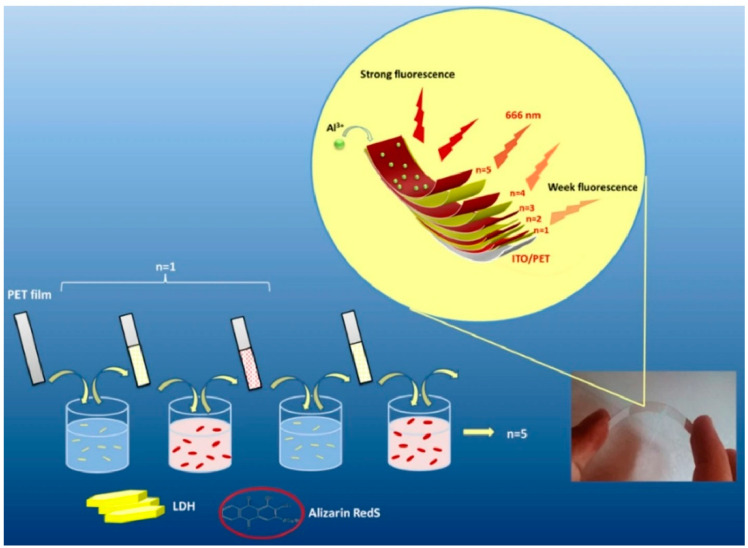
Electrochemical solid-state sensors based on flexible ITO-coated polyethylene terephthalate (PET) constructed with the sensing element consist of Mg–Al LDH nanoplatelets and Alizarin Red S (ARS upon LbL assembly). Reproduced under terms of the CC-BY license [318]. Copyright 2020 Springer-Nature.

Investigations have been conducted on layered materials, such as layered double hydroxide (LDH) [316,317], in view of their diverse functionalities, including dye intercalation. For example, the development of an Al^3+^ detection sensor by LbL assembly of LDH and dyes has been reported by Rouhani and co-workers (Figure 12) [318]. Despite the fact that toxic Al^3+^ ions have serious effects on the human nervous system, such as those related to Alzheimer’s disease, the development of solid electrodes for sensor-based Al^3+^ detection is not necessarily sufficient. In this study, electrochemical solid-state sensors based on flexible ITO-coated polyethylene terephthalate (PET) substrates were designed and constructed. The sensing element consists of Mg–Al LDH nanoplatelets and Alizarin Red S (ARS), fabricated by LbL assembly. ARS, an electroactive organic unit present in the layered (ARS/LDHs)n matrix, can detect the electrochemical changes of the Al chelating system when exposed to the target Al^3+^. The LbL sensor exhibits high sensitivity and selectivity in detecting Al^3+^ through both electrochemical and optical methods, thereby combining the characteristics of cyclic voltammetry and fluorescence-based optical methods. This system has the potential to serve as a methodology for biotechnological diagnostics.

### 2.12. Metal–Organic Frameworks (MOFs)

**Figure 13:**
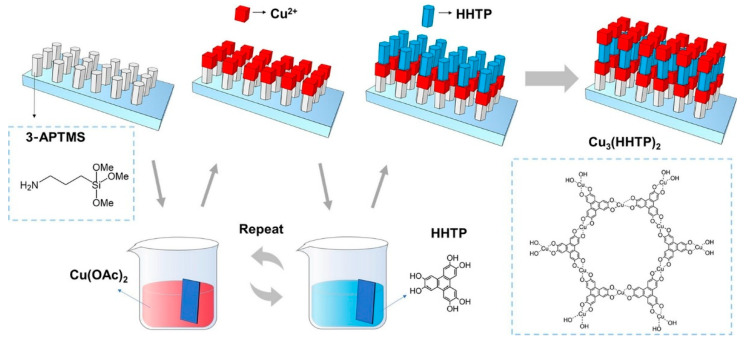
Fabrication of d Cu_3_(HHTP)_2_ MOF thin films of controllable thickness on conductive fluorine-doped tin oxide (FTO) glass surfaces by LbL assembly. Reprinted with permission from [327]. Copyright 2021 Elsevier.

Metal–organic frameworks (MOFs) or coordination polymers have been the focus of significant research in recent years [319,320,321,322,323] due to their ability to be fabricated by bottom-up processes, enabling the creation of precise porous structures. The synthesis of two-dimensional (2D) MOFs at interfaces and their assembly into layer-by-layer structures has also garnered considerable attention [324,325,326]. Among them, triphenylene-based MOFs are poised to play a crucial role in sensors, batteries, supercapacitors, catalysis, and optoelectronics. Li, Wang, and co-workers have fabricated Cu_3_(HHTP)_2_ MOF thin films of a controllable thickness on conductive fluorine-doped tin oxide (FTO) glass surfaces by LbL assembly at room temperature (Figure 13) [327]. The fluorine-doped tin oxide substrate was pretreated to immobilize amino groups on the surface. Cu_3_(HHTP)_2_ films of varying thicknesses were then prepared by in situ self-assembly on fluorine-doped tin oxide substrates that had been modified via layer-by-layer immersion in Cu^2+^ and 2,3,6,7,10,11-hexahydroxytriphenylene (HHTP) solutions. The investigation of the obtained Cu_3_(HHTP)_2_ films was undertaken with a view to their electrochromic behavior, the results of which demonstrated a fast color change rate, a high photomodulation range, and a high coloring efficiency. The advantageous properties of the porous structure, high electrochemical redox activity, and low contact resistance with the electrolyte of the Cu_3_(HHTP)_2_ film electrode are responsible for its fast coloring rate, good photomodulation range, and high coloring efficiency. The results obtained demonstrate the potential application of MOF LbL films in the electrochromic field, with a wide range of applications anticipated in semiconductors and optoelectronics.

### 2.13. Covalent Organic Frameworks (COFs)

**Figure 14:**
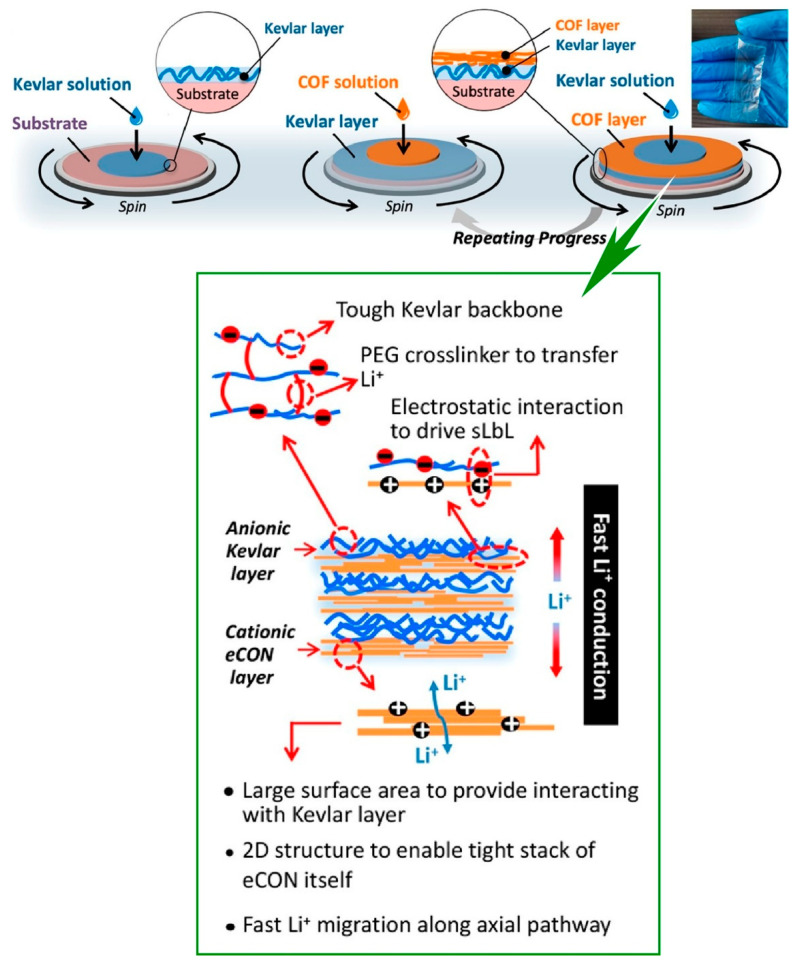
Fabrication of heterolayered Kevlar/COF composite membrane through a bottom-up spin LbL assembly technique: fabrication outline (**top**) and functional advantages (**bottom**). Reprinted with permission from [333]. Copyright 2020 American Chemical Society.

Covalent organic frameworks (COFs), which are composed of organic units linked through covalent bonds to form porous structures, have also garnered significant interest [328,329,330]. The synthesis of multilayered COF structures has been reported [331,332], with particular attention being directed towards their potential applications in the energy sector. Specifically, there is a significant demand for lightweight membranes with a thickness of less than tens of microns, with the objective of maximizing the energy density of solid-state batteries for next-generation lithium-based batteries. Lu, Chen, and their colleagues have developed a heterolayered Kevlar/COF composite membrane through a bottom-up spin LbL assembly technique, which enables precise control over the membrane structure and thickness (Figure 14) [333]. The unitized membrane was fabricated by means of spinning LbL assembly and was molded to a precisely controlled thickness. The Kevlar component provides structural advantages in terms of mechanical strength, and the layered 2D COF sheets provide fast Li^+^ conductivity and can be tightly interlocked with Kevlar through electrostatic interactions. The synthesis of a robust and Li^+^-conductive electrolyte film was enabled by the significantly enhanced chemical/mechanical interactions between the crosslinked Kevlar and the conductive 2D–COF components. Furthermore, the fast directional diffusion of Li^+^ was achieved locally along the channel direction, perpendicular to the assembled 2D COFs, which function as excellent ionic conductors and facilitate the release of free Li^+^ species. Through the LbL assembly of COFs, this study proposes a novel concept of an ultrathin, ultrastrong, and conductive solid composite electrolyte based on heterolayer architecture for highly safe solid-state lithium metal batteries. The development of such multifunctional membranes through layer-by-layer nanoarchitectonics paves the way for the development of high energy density solid-state lithium metal batteries in the future.

### 2.14. Conducting Polymers with MXenes

**Figure 15:**
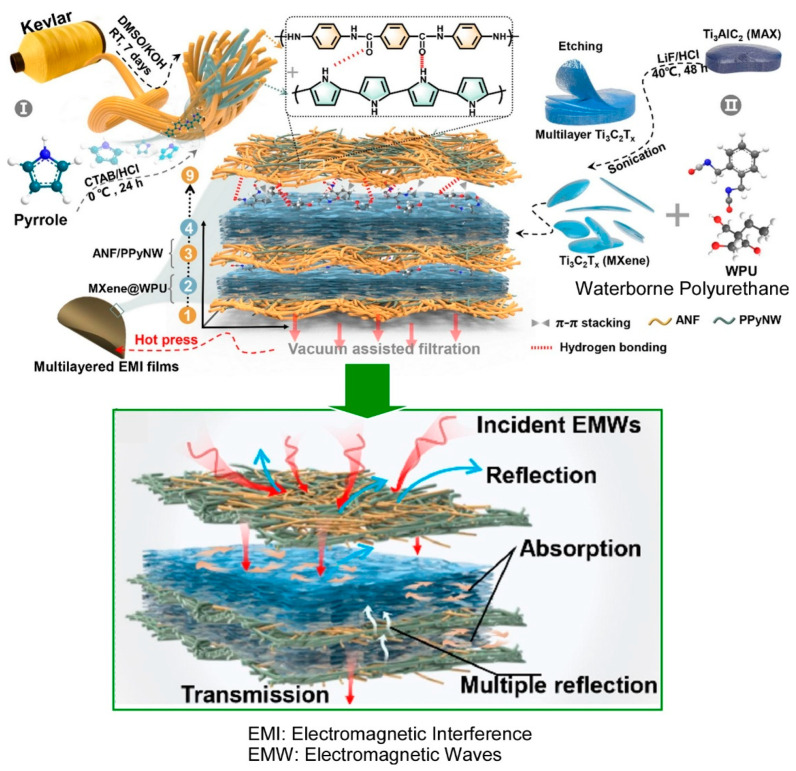
Fabrication of a flexible multilayer electromagnetic shielding and interference shielding composite film based on alternately layering with aramid nanofibers/polypyrrole nanowires and Ti_3_C_2_T_x_ reinforced with water-based polyurethane: film construction (**top**) and representative properties (**bottom**). Reprinted with permission from [343]. Copyright 2022 Elsevier.

Conductive polymers have a valuable role in device-oriented functional research [334,335,336,337]. Conductive polymers are also active targets of LbL assembly [338,339,340]. MXene, a 2D material, has also garnered significant attention in numerous research domains [341,342]. In particular, high-performance conductive polymer nanocomposites containing 2D MXene have attracted significant interest in the field of electromagnetic shielding and interference in flexible electronics. Zhang and colleagues have fabricated a flexible multilayer electromagnetic shielding and interference shielding composite film based on MXene and intrinsically conductive polymer (Figure 15) [343]. The multilayer film, which is alternately layered with aramid nanofibers/polypyrrole nanowires and Ti_3_C_2_T_x_ reinforced with water-based polyurethane, was fabricated by a simple alternating vacuum filtration method. The fabricated layer-by-layer thin film demonstrated high electrical conductivity and exhibited an excellent electromagnetic shielding effect. The conductive aramid nanofiber/polypyrrole nanowire layer, which functions as a protective layer, is constructed by a continuous polypyrrole conductive network, which possesses desirable properties such as flexibility, light weight, and high electrical conductivity. The employment of layer-by-layer nanoarchitectonics facilitates the integration of materials with disparate properties and functions, thereby enabling the creation of multifunctional materials.

### 2.15. DNA-Related Materials

**Figure 16:**
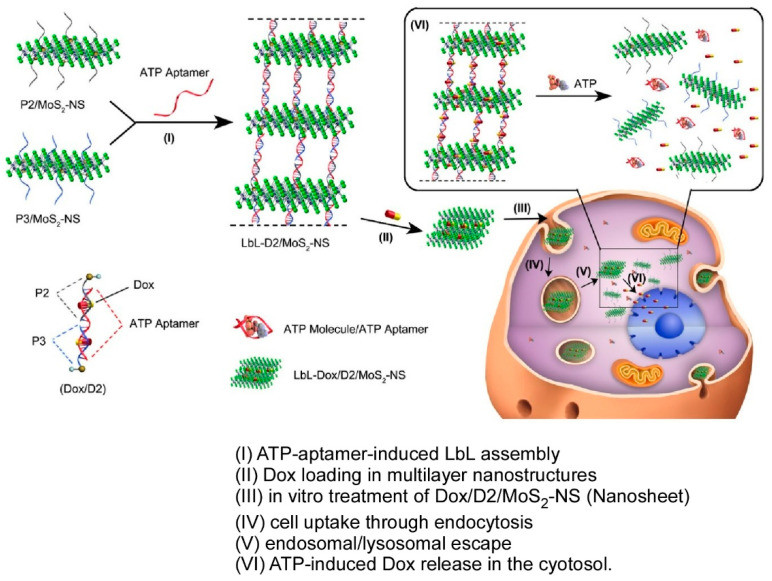
A system in which LbL self-assembled layered MoS_2_ superstructures carrying anticancer drugs with the property of autonomously disassembling in response to the enhanced ATP metabolism of cancer cells. Reprinted with permission from [350]. Copyright 2017 American Chemical Society.

It has been established that DNA exerts various biological functions as genes and corresponding biological properties [344,345]. In addition, it has also been studied as a material for nanostructures, such as DNA origami, due to its high complimentary recognition [346,347]. DNA is a polyelectrolyte, which makes it a very suitable material for LbL assembly based on electrostatic interactions [348,349]. Indeed, many studies have been conducted on the LbL assembly of DNA. Furthermore, DNA can be used as a structural recognition material to construct more advanced layered structures by hybridizing them with other functional materials. Leong and co-workers have constructed a system in which LbL self-assembled layered MoS_2_ superstructures carrying anticancer drugs can be controlled by sequence-based DNA oligonucleotide interactions (Figure 16) [350]. In the first stage of the process, the authors functionalized MoS_2_ nanosheets (MoS_2_-NS) with DNA oligonucleotides-bearing thiol terminal groups (DNA/MoS_2_-NS) via strong binding to sulfur atom defect vacancies on the MoS_2_ surface. To further assemble the structure into a higher-order DNA/MoS_2_-NS superstructure, they incorporated a linker aptamer that induces interlayer assembly. This superstructure possesses the property of autonomously disassembling in response to the enhanced ATP metabolism of cancer cells, and the linker utilized contains an ATP aptamer sequence, resulting in the disassembly of the superstructure into the original individual nanosheets in response to ATP stimulation. Once inside the cell, small ATP molecules access the stacked nanosheets and bind to the ATP aptamer linker, triggering a conformational change in the ATP aptamer. The dissociation of the linker also leads to the disassembly of the higher-order structure, resulting in the release of the anticancer drug Dox, which was initially incorporated. Through the precise modification of the oligonucleotide length, this valuable gap can be tuned with angstrom precision, thereby enabling the control of the selectivity of the anticancer drug. The combination of DNA specificity and the 2D flatness of MoS_2_ gives rise to a designable autonomous stimuli-responsive drug delivery system based on multilayered DNA/MoS_2_-NS. This layer-by-layer nanoarchitectonic represents a significant step forward in the field of nanomedicine, with the potential to enhance stimuli-responsive drug release systems for targeted chemotherapy. Furthermore, it is anticipated that this technology will find application in other domains of nanotechnology.

### 2.16. Polysaccharides

**Figure 17:**
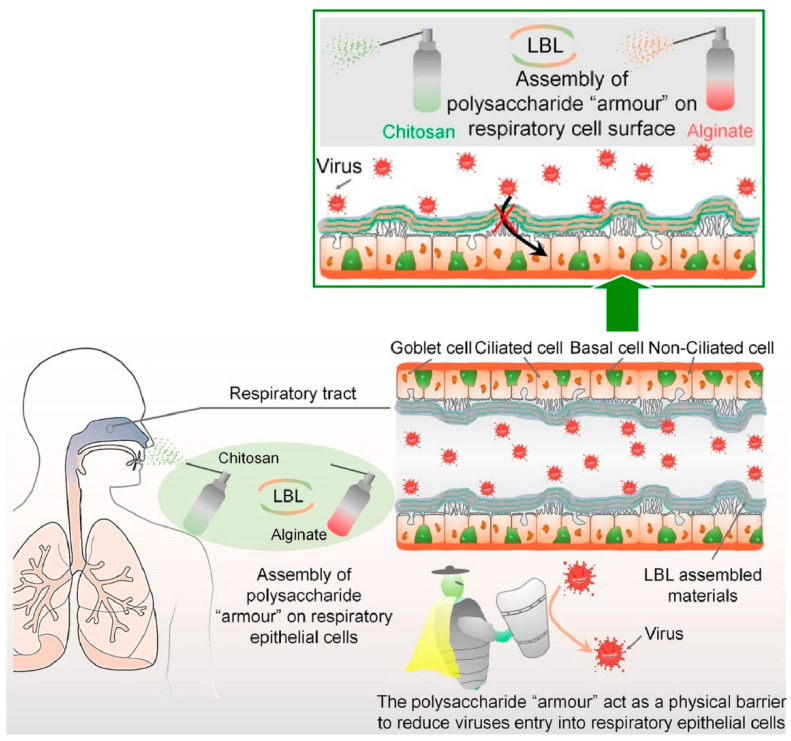
A nasal spray method to form a polysaccharide armor on the cell surface by spray-based LbL assembly method to minimize the risk of viral infection using positively charged chitosan and negatively charged alginate. Reprinted with permission from [355]. Copyright 2022 American Chemical Society.

Polysaccharides have been identified as key macromolecules in the context of addressing biological challenges, including viral infections, and have thus been the focus of considerable research [351,352]. The presence of charges in certain polysaccharides and related macromolecules renders them suitable for utilization as components in LbL assembly [353,354]. The development of LbL membranes composed of polysaccharides has the potential to provide solutions to significant biomedical challenges. Airborne pathogens, such as severe acute respiratory syndrome coronavirus 2 (SARS-CoV-2), which is spreading worldwide, cause global pandemics by infection through the respiratory system. The LbL assembly of polysaccharides has been proposed as a means of protecting individuals from future pandemics. Fan, Zhu, and co-workers developed a nasal spray that forms a polysaccharide armor on the cell surface by a spray-based LbL assembly method to minimize the risk of viral infection (Figure 17) [355]. The method involves the administration of a nasal spray comprising two distinct components, as follows: positively charged chitosan and negatively charged alginate. Upon inhalation, these polysaccharides assemble in a sequential manner to create a polysaccharide “armor” layer on the cell surface, thereby impeding the entry of viruses into the cells. This approach exhibits minimal cell toxicity and confers upon cells the capacity to resist infection by adenovirus and the SARS-CoV-2 pseudovirus. The positively charged chitosan is retained in the nasal cavity for an extended period, thereby ensuring the stability of the polysaccharide barrier and making it an effective absorption enhancer. In comparison to negatively charged xylitol and carrageenan, it is more promising in terms of its resistance to mucociliary clearance. It is also noteworthy that it uses materials that have been approved by the Food and Drug Administration (FDA), in contrast to regular nasal sprays which contain drugs, antibodies, bioactive proteins, or cellular components. The LbL assembly of the polysaccharide armor had only a minor effect on cell proliferation and provided long-term protection against viral infection. Consequently, it may be employed as a standalone or supplementary preventive measure to address the ongoing challenges posed by the pandemic.

### 2.17. Nanocellulose Materials

**Figure 18:**
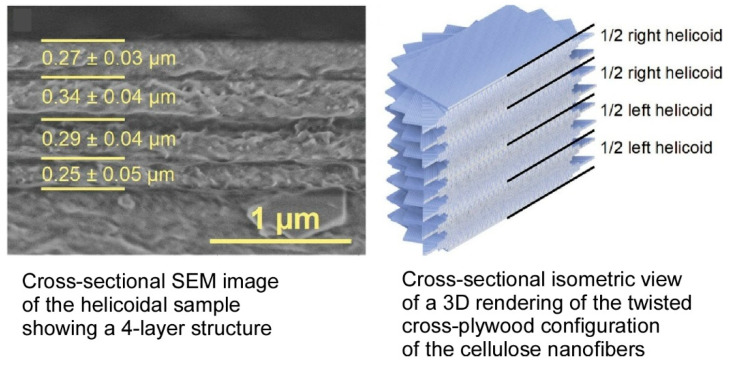
An additive manufacturing process for spray-assisted LbL assembly of cellulose nanofibrils: (**left**) a cross-sectional SEM image of a helical sample, illustrating a four-layer structure that corresponds to four half-pitch values of the helix; (**right**) the 3D model created by stacking 10-nm-thick parallel cylinder layers following the same alignment sequence. Reproduced under terms of the CC-BY license [359]. Copyright 2024 Wiley-VCH.

Cellulosic nanocomposites have attracted significant attention in recent years due to their potential for the development of sustainable functional materials [356,357,358]. However, achieving precise structural control at the submicron scale remains challenging, and the full potential of fibrous nanocomposites is yet to be realized. In addressing this, Decher, Houérou, Felix, and their colleagues have developed an additive manufacturing process for spray-assisted LbL assembly of cellulose nanofibrils, enabling the production of thin films with helical alignment [359]. This process involves the rational selection of the alignment direction of each cellulose layer, allowing for the straightforward adjustment of handedness and pitch of the chiral structure through the deliberate selection of parameters such as the number of consecutive cellulose layers sprayed in the same direction and the rotation angle between consecutive layer stacks. Figure 18 presents a cross-sectional SEM image of a helical sample, illustrating a four-layer structure that corresponds to four half-pitch values of the helix. The figure also showcases a cross-sectional isometric view of a 3D rendering of the twisted cross-plywood configuration of cellulose nanofibrils in the helical film. The 3D model was created by stacking 10-nm-thick parallel cylinder layers following the same alignment sequence as the experimental sample. The handedness and pitch of the helical arrangement of cellulose nanofibrils can be controlled by rationally selecting the spray direction, the in-plane rotation angle between layer assemblies, and the number of layers oriented in the same direction. This methodology is of significant value, as it facilitates the preparation of complex nanocomposite structures comprising cellulose nanofibers with diverse nanoscale substructures, which hold considerable potential as eco-friendly alternatives to petroleum-derived raw materials. The additive manufacturing method presented in this study can also be readily transferred to other nanofiber components, for example, to enhance the filtering efficiency of circularly polarized light. Such multifunctional bio-based composites have the potential to compete in future applications, such as high-performance composites, protective coatings, optical filters, and flexible displays. This approach addresses the environmental and societal imperatives for sustainable, high-performance products that are not petroleum-based.

### 2.18. Peptides

**Figure 19:**
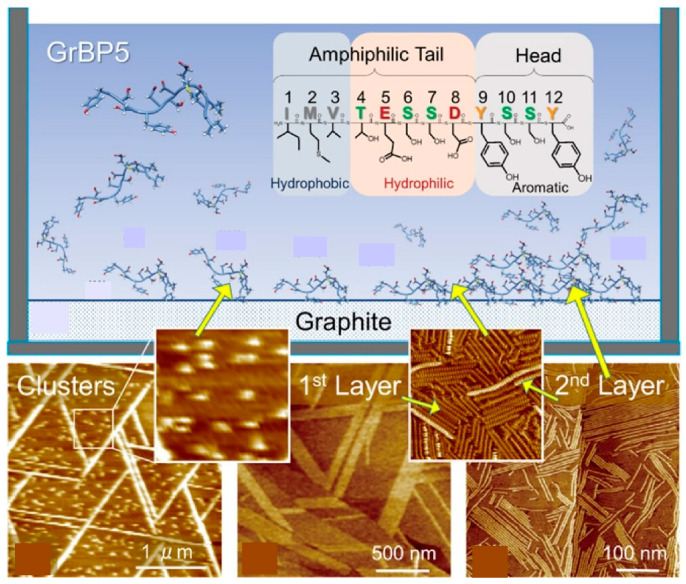
Self-assembly process of the initial two layers of graphite-bound dodecapeptide GrBP5 (graphite-binding peptide 5 (GrBP5)) on a highly oriented pyrolytic graphite surface in an aqueous solution through a series of molecular interactions, including conformational states, molecule–substrate interactions, and intermolecular interactions. Reprinted with permission from [363]. Copyright 2023 American Chemical Society.

The self-assembly of peptides has been a subject of study in various bio-related research fields [360,361,362]. Structure-controlled peptide molecules exhibit highly defined assembly forms, and their layered organization is also of interest. The mechanism by which short peptides self-assemble into two- and three-dimensional structures is of great interest in the formation of crystalline biomolecular systems and their practical applications. Exploration of the self-assembly of solid-bound peptides on surfaces would be a fruitful area of research to address these issues. In their seminal study, Yurtsever, Sun, Sarikaya, and their co-workers utilized the graphite-bound dodecapeptide GrBP5 (graphite-binding peptide 5 (GrBP5)) to investigate the self-assembly process of the initial two layers on a highly oriented pyrolytic graphite surface in an aqueous solution (see Figure 19) [363]. The structural observation was conducted in situ by means of frequency modulation atomic force microscopy. The dodecapeptide GrBP5 is an amphiphilic biomolecule with three chemical domains that guide the molecule to self-assemble on the surface and assemble into layers through a series of molecular interactions, including conformational states, molecule–substrate interactions, and intermolecular interactions. First, the first layer is uniformly formed, producing self-assembled crystals with a lattice structure in contact with the underlying graphite. The formation of the second layer is non-uniform, primarily due to the crystal defects present in the first layer, and progresses in a sequential manner to yield a self-assembled structure comprising a transient crystalline phase. The peptide molecular lattice adopts a distinctive orientation relative to the underlying graphene lattice, suggesting the potential for the establishment of a coherent bio/nano electronic interface. This phenomenon is of significance for both scientific understanding and the construction of coherent bio/nano hybrid interfaces. A comprehensive understanding of the intricate surface phenomena during the peptide self-assembly process is instrumental for the development of hybrid surface technologies, protein and peptide arrays, bioelectronics, and biomolecular logic devices. This understanding can provide fundamental guidance for the design of genetically encoded and controlled organized nanostructures at the molecular scale.

### 2.19. Proteins

**Figure 20:**
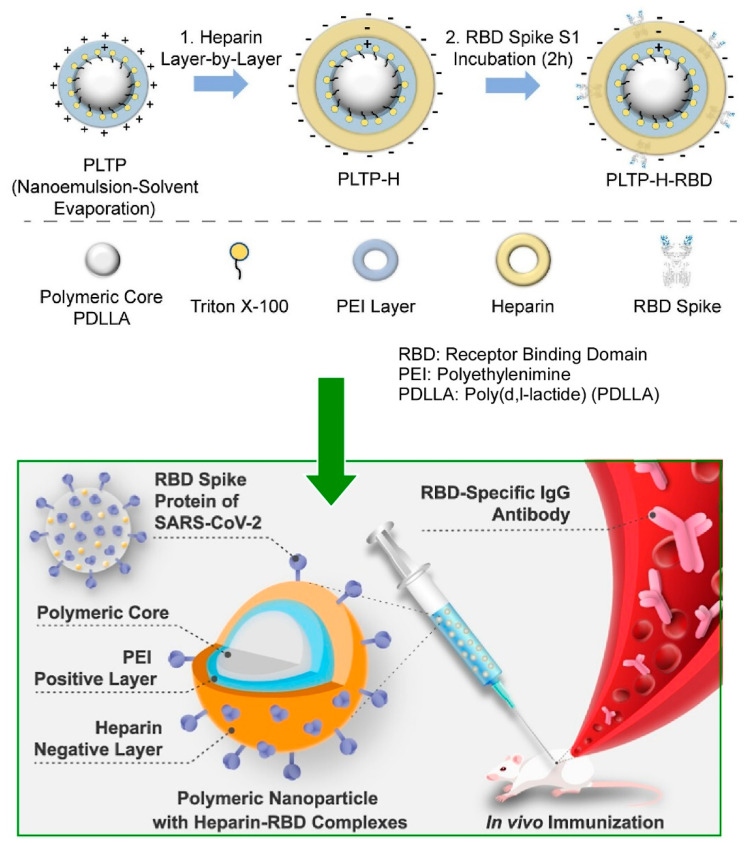
Versatile multilayer polymer particles as a vaccine approach against SARS-CoV-2, based on binding a recombinant form of the receptor-binding domain (RBD) antigen of the SARS-CoV-2 spike protein: fabrication outline (**top**) and biological usage (**bottom**). Reprinted with permission from [372]. Copyright 2024 American Chemical Society.

Proteins are a highly functional class of biomolecule that have been the focus of extensive research in numerous fields [364,365,366,367]. Many proteins possess surface charges and are frequently utilized as materials for LbL assembly through electrostatic interactions [368,369]. LbL assembly via biospecific recognition functions has also been reported [370,371]. The creation of medical materials is achieved by immobilizing specific proteins on polymer particles and other materials through LbL assembly. Such multilayer polymer particles have been shown to function as carriers for compounds with controlled release, triggered by layer degradation, and for facilitating drug transport through the bloodstream and tissues. In a recent study, Catalani and colleagues reported the use of versatile multilayer polymer particles as a vaccine approach against SARS-CoV-2, based on binding a recombinant form of the receptor-binding domain (RBD) antigen of the SARS-CoV-2 spike protein (Figure 20) [372]. These particles have the capacity to carry and release the RBD region. The core of the particles is composed of poly(d,l-lactide), coated with Triton X-100 and polyethyleneimine, which has been functionalized layer by layer with heparin (PLTP-H). This has been prepared by the nanoemulsion/solvent evaporation method, and the nanoparticles have then been added with a negative outer layer to immobilize the recombinant RBD protein produced in mammalian cells by the LbL approach. The recombinant RBD is thus anchored to the nanoparticles by the negative outer layer (PLTP-H-RBD). The antigen-loaded nanoparticles have been shown to enhance specific serum IgG levels against the recombinant protein. Furthermore, mice immunized with PLTP-H-RBD, in contrast to animals immunized with the same amount of uncomplexed antigen, exhibited enhanced levels of virus-neutralizing antibodies and protection against a lethal challenge with the SARS-CoV-2 Wuhan strain. The functionalized nanoparticles obtained in this study played a suitable adjuvant role and conferred a protective immune response against SARS-CoV-2. This layered nanoarchitectonics provides a valuable technological foundation for the development of subunit vaccines based on recombinant proteins.

### 2.20. Lipid Bilayers

**Figure 21:**
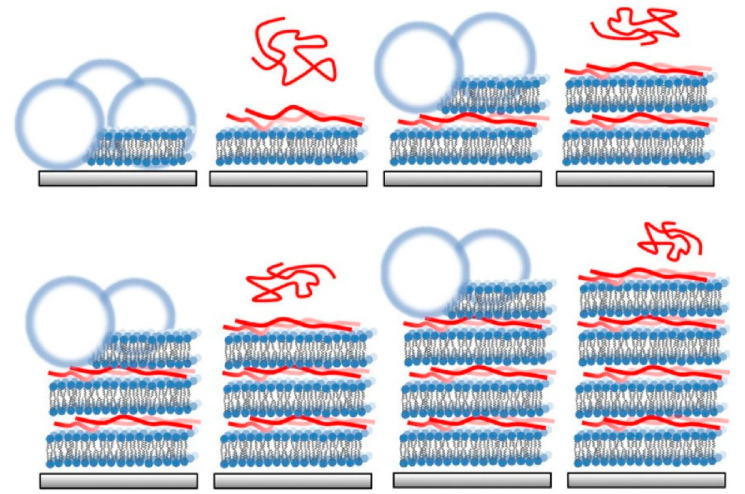
An LbL assembly using poly-L-lysine as an electrostatic polymer linker to form lipid multilayers on a supported lipid bilayer via lipid vesicle rupture, where positively charged poly-L-lysine is adsorbed onto a negatively charged base-supported lipid bilayer. Reproduced under terms of the CC-BY license [379]. Copyright 2016 American Chemical Society.

Lipid bilayer membranes represent the predominant components of cell membranes, and the functionality of model membranes and endoplasmic reticulum structures (e.g., liposomes, vesicles) has been the subject of investigation [373,374,375]. Such membrane structures can also be targeted by LbL assembly. For instance, artificial lipid bilayer structures [376,377] and Langmuir–Blodgett films [378] that mimic them have been stacked by LbL. Furthermore, endoplasmic reticulum-mimicking cell-like lipid bilayer vesicles have been assembled in multilayers by LbL assembly. Jeuken and co-workers reported an LbL assembly that uses poly-L-lysine as an electrostatic polymer linker to form lipid multilayers on a supported lipid bilayer via lipid vesicle rupture (Figure 21) [379]. Poly-L-lysine, which is positively charged, is adsorbed onto a negatively charged base-supported lipid bilayer until it is saturated. Excess poly-L-lysine is then washed away, and a second membrane is formed on the resulting positively charged surface via a solution of negatively charged lipid vesicles. This process is repeated to form layer-by-layer nanoarchitectonics of lipid bilayer membranes. This process is repeated to form densely packed layers through varying the buffer pH and poly-L-lysine chain length. The incorporation of functional components, such as proteins, provides a straightforward method for creating complex membrane structures found in nature, and a platform for a deeper understanding of biological processes that can be applied in fields such as energy production and biosensing. This not only provides a system for future potential technologies, but also a system for understanding fundamental biological processes at the membrane–membrane interface.

### 2.21. Photosystems

**Figure 22:**
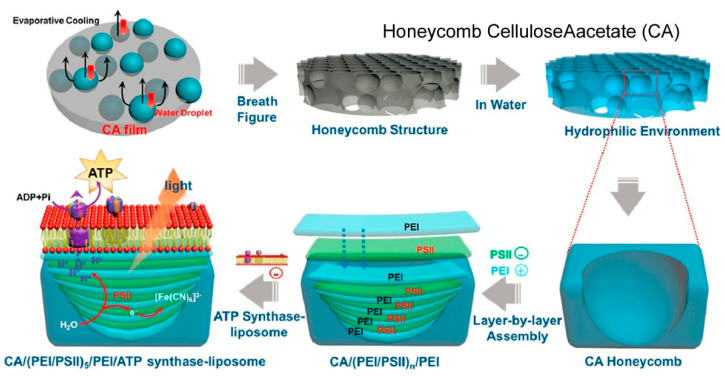
An artificially designed honeycomb multilayer structure for photophosphorylation by a multilayered photosystem II (PSII)-ATP synthase–liposome system through LbL assembly, where, under light irradiation, photosystem II splits water into protons, thereby generating a proton gradient that is utilized by ATP synthase to synthesize ATP. Reprinted with permission from [384]. Copyright 2018 American Chemical Society.

It has been demonstrated that more complex and advanced biological systems can also be organized through LbL assembly [380,381]. One of the primary objectives of this process is to emulate the photosynthetic process. Photosynthesis, a process integral to plant life, occurs within the thylakoids of plant cells, which exhibit a characteristic stacking organization [382,383]. Within this structure, the processes of light harvesting, water splitting, and adenosine triphosphate (ATP) production are facilitated. The stacking structure plays a pivotal role in facilitating the exchange of substances during photosynthesis with exceptional efficiency and minimal energy expenditure. Xie, Yang, Li, and co-workers have reported an artificially designed honeycomb multilayer structure for photophosphorylation (Figure 22) [384]. For this, a multilayered photosystem II (PSII)-ATP synthase–liposome system was created by LbL assembly. This structure facilitates a three-dimensional distribution of photosystem II and ATP synthase. Under light irradiation, photosystem II splits water into protons, thereby generating a proton gradient that is utilized by ATP synthase to synthesize ATP. The integration of photosystem II and ATP synthase, according to their natural structure and three-dimensional distribution, has been demonstrated to be a successful approach for generating ATP with high efficiency. The construction of an integrated photosystem II and ATP synthase system that mimics the photosynthetic grana structure has been demonstrated by multilayer structures assembled via LbL assembly. This approach, termed layer-by-layer nanoarchitectonics, has emerged as a promising strategy for the assembly of bioinspired systems with complicated structures, offering a simple and efficient method for the construction of complex biological structures.

### 2.22. Virus Particles

The utilization of charged viruses as charged polyelectrolytes in multilayer film construction has also been demonstrated. Möhwald and colleagues selected the carnation mottle virus as an example of such a charged particle and demonstrated the assembly of a molecular thin film by layer-by-layer adsorption with polycation layers [385]. This plant RNA-containing virus possesses a spherical shape with an outer diameter of 340 Å and is negatively charged due to the RNA incorporated in the viral shell. The virus crystals were dissolved in a buffer and LbL assembly was performed with positively charged polyallylamine. This approach demonstrates the potential for viruses to serve as components in layer-by-layer nanoarchitectonics.

### 2.23. Cells and Tissues

**Figure 23:**
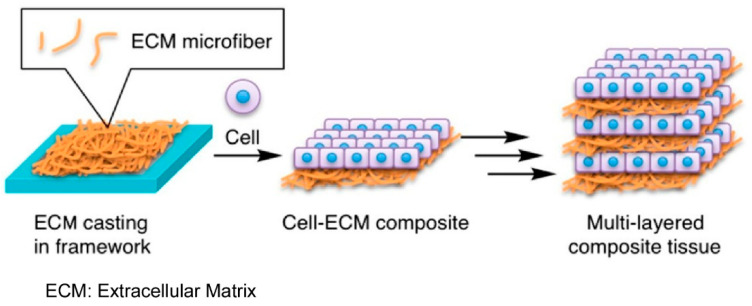
A multilayered tissue consisting of an extracellular matrix layer and a cell layer by assembling cell-seeded extracellular matrix papers. Reprinted with permission from [392]. Copyright 2019 American Chemical Society.

The organization of living cells has attracted significant attention in both medical research [386,387] and the development of biofunctional structures [388,389]. The in vitro construction of highly organized 3D tissues represents a significant challenge in the field of tissue engineering. LbL assembly is a useful method for achieving such targets [390,391]. Specifically, Nakatsuji and Matsusaki fabricated a multilayered tissue consisting of an extracellular matrix layer and a cell layer by assembling cell-seeded extracellular matrix papers (Figure 23) [392]. In this study, they constructed a multilayered composite tissue based on the preparation of natural elastin- and collagen-based extracellular matrix papers. To prepare the extracellular matrix paper, they microfibrillated natural elastin to create a dispersion of elastin microfibers. The extracellular matrix paper was fabricated by casting the extracellular matrix dispersion on a silicone rubber framework. The resultant extracellular matrix paper exhibited high tensile strength, cell adhesion properties, and stacking properties for preparing multilayered tissues, depending on the mixing ratio of elastin microfibers and type I collagen. A notable advantage of utilizing a natural insoluble extracellular matrix in the preparation of the extracellular matrix paper is that chemical denaturation is not a prerequisite. The stability of the extracellular matrix paper under physiological conditions, coupled with its biocompatibility, renders it a suitable candidate for use as a cell scaffold. Paper-like scaffolds are considered to be excellent materials for the construction of 3D tissues due to their high permeability and cell migration capacity. The method presented here allows for control over the thickness and composition of the extracellular matrix in multilayered 3D tissues. This attribute is beneficial for constructing normal and diseased tissue models. The potential of these 3D tissues to serve as a model for evaluating cell migration and cell–cell and cell–extracellular matrix interactions in multilayered biological tissues, such as blood vessel walls, is a significant advantage.

## 3. Conclusions and Perspectives

This review presents a variety of examples of layer-by-layer nanoarchitectonics. A notable feature is the diversity of components and materials used in LbL assemblies. The examples presented in this paper include quantum dots, nanoparticles, nanocrystals, nanowires, nanotubes, g-C_3_N_4_, graphene oxide, MXene, nanosheets, zeolites, nanoporous materials, sol–gel materials, layered double hydroxides, metal–organic frameworks (MOFs), covalent organic frameworks (COFs), conducting polymers, dyes, DNAs, polysaccharides, nanocelluloses, peptides, proteins, lipid bilayers, photosystems, viruses, living cells, and tissues. It should be noted that this is not an exhaustive list, and there are many other possibilities. This paper has focused on examples of LbL assemblies, but it should be noted that there are different methods for creating multilayer structures, such as the LB method. The result is that a very large number of materials can be organized into functional structural materials through layered nanoarchitectonics.

The listed examples are manifold. It can be noticed that the characteristics and trends are not dependent on any one substance, but rather are characterized by common characteristics being used for many substances. This indicates that the LbL assembly method has evolved to adapt to many materials and methods. Starting with simple macromolecular layered structures, the introduction of biomaterials, nanomaterials, and even living cells have been layered. This is made possible by the fundamental principle of LbL assembly, which is to use interactions that universally exist between molecules and materials. In addition, new methods have been introduced, such as composite organization of many materials, stabilization by covalent bonding, and patterning to a specific structure. The universality of the principles and the freedom to incorporate new concepts are key to the development of LbL assembly. Its diverse possibilities create new challenges. We will discuss them briefly below.

Finally, the future prospects of this layer-by-layer nanoarchitecture must be considered. The strength of LbL assembly lies not only in the diversity of materials, but also in the simplicity of the method. It is often assumed that simple layered structures can be easily created, but the advantage of being able to create them in this way should be used to develop more complex structures. The assembly of diverse functional components into complex sequences and integrated structures is a likely and attractive outcome of LbL assembly. The organization of functional structures in living organisms is rational, with advanced functions exerted through the cooperation of functions and the transmission of signals. The direction for layer-by-layer nanoarchitectonics should be to assemble complex functional structures that cannot be achieved by normal self-assembly. The aim should be to express high-level functions similar to those observed in living organisms. It is important to note, however, that complexity beyond a certain threshold may not be feasible based solely on fundamental principles and the experience of researchers. Fortunately, there have been rapid advances in artificial intelligence, with machine learning being actively explored in the fields of chemical and materials engineering [393,394,395,396,397] and materials informatics [398,399,400]. The importance of integrating nanoarchitectonics and materials informatics has also been discussed [401,402]. The future direction of layer-by-layer nanoarchitectonics will be to use artificial intelligence to construct functional systems that rival biological functions. In fact, the application of machine learning to the prediction and evaluation of nanoscale surface topology in LbL assembly has been reported [403].

Another future direction is the path to practical application, which is facilitated by the simplicity of LbL assembly, enabling low-cost performance. This is a significant advantage for the practical application of functional thin films, as LbL assembly can be performed with minimal equipment, such as tweezers and a beaker. The incorporation of LbL assembly into an immersion apparatus has led to the development of an automatic film-forming apparatus. Another noteworthy aspect is the development of the spray method, which facilitates the rapid production of layered films. Examples of layering on meter-sized substrates using a roll-dipping device and on the surface of a car body using a car wash machine have been documented [404]. It is expected that significant progress will be made in this area as the industrial world focuses on layer-by-layer nanoarchitecture and pursues practical applications. It is expected that such scientific literature will act as stimuli and triggers for the subsequent industrialization of the field.
